# Infant feeding practices and determinants of poor breastfeeding behavior in Kinshasa, Democratic Republic of Congo: a descriptive study

**DOI:** 10.1186/1746-4358-8-11

**Published:** 2013-10-01

**Authors:** Marcel Yotebieng, Jean Lambert Chalachala, Miriam Labbok, Frieda Behets

**Affiliations:** 1 College of Public Health, Division of Epidemiology, The Ohio State University, Columbus, OH, USA; 2 Department of Epidemiology, The University of North Carolina at Chapel Hill, Chapel Hill, NC, USA; 3 School of Public Health, The University of Kinshasa, Kinshasa, DR, Congo; 4 Carolina Global Breastfeeding Institute, Department of Maternal and Child Health, The University of North Carolina at Chapel Hill, Chapel Hill NC, USA; 5 School of Medicine, The University of North Carolina at Chapel Hill, Chapel Hill, NC, USA

**Keywords:** Breastfeeding, Exclusive breastfeeding, Infant feeding practices, Kinshasa, DR Congo

## Abstract

**Background:**

Although breastfeeding is almost universally accepted in the Democratic Republic (DR) of Congo, by the age of 2 to 3 months 65% of children are receiving something other than human milk. We sought to describe the infant feeding practices and determinants of suboptimal breastfeeding behaviors in DR Congo.

**Methods:**

Survey questionnaire administered to mothers of infants aged ≤ 6 months and healthcare providers who were recruited consecutively at six selected primary health care facilities in Kinshasa, the capital.

**Results:**

All 66 mothers interviewed were breastfeeding. Before initiating breastfeeding, 23 gave their infants something other than their milk, including: sugar water (16) or water (2). During the twenty-four hours prior to interview, 26 (39%) infants were exclusively breastfed (EBF), whereas 18 (27%), 12 (18%), and 10 (15%) received water, tea, formula, or porridge, respectively, in addition to human milk. The main reasons for water supplementation included “heat” and cultural beliefs that water is needed for proper digestion of human milk. The main reason for formula supplementation was the impression that the baby was not getting enough milk; and for porridge supplementation, the belief that the child was old enough to start complementary food. Virtually all mothers reported that breastfeeding was discussed during antenatal clinic visit and half reported receiving help regarding breastfeeding from a health provider either after birth or during well-child clinic visit. Despite a median of at least 14 years of experience in these facilities, healthcare workers surveyed had little to no formal training on how to support breastfeeding and inadequate breastfeeding-related knowledge and skills. The facilities lacked any written policy about breastfeeding.

**Conclusion:**

Addressing cultural beliefs, training healthcare providers adequately on breastfeeding support skills, and providing structured breastfeeding support after maternity discharge is needed to promote EBF in the DR Congo.

## Background

Millennium Development Goal (MDG) 4 calls for a two-third reduction in the under-five mortality rate by 2015. Between 1990 and 2010, global deaths among children under 5 years of age declined from over 12 million to 7.6 million [1]. However, according to *Countdown to 2015*, an organization that monitors progress towards reaching MDG 4, of 74 focus countries with available data for 2012 report, only 23 were on track to achieve the goal and 13 had made no progress. All but one (Haiti) of the countries that had made no progress are in Sub-Saharan Africa [2].

The Democratic Republic (DR) of Congo is one of the 13 countries which has seen no progress towards MDG 4. It bears the third largest burden of child deaths worldwide [3] and its under-five mortality rate has remained high: from 180 for every 1000 live births in 1990 to 170 in 2010. Although these deaths are the result of a web of complex determinants [4], there is enough evidence to believe that breastfeeding practices play a major role in the extremely high infant mortality in the DR Congo. First, results from the 2007 Demographic and Health Survey (DHS) [5] show that of the 9.2% of infants who die before their first birthday in DR Congo, 4.2% die during the neonatal period and the remaining 5% between 1 and 12 months. Second, of the 116 out of 1000 babies born alive in 2010 in DR Congo who survived through the first 28 days and subsequently died before their fifth birthday, 20 died from diarrhea and 23 from pneumonia, only malaria claim more under-5 lives (28) while AIDS accounts for only 2 [3]. More deaths in the postnatal period and the predominant role of diarrhea, pneumonia and malaria suggest that factors behind these deaths are to be found among other sources in the feeding practices. In fact, by the age of 6 months, more than 10% of children in DRC are already stunted, virtually 15% are underweight-for-age and approximately the same percentage emaciated [5].

These data have to be understood in the context of relatively high utilization of primary health services and high rates of breastfeeding initiation. Despite the challenges to accessing health care in DR Congo, DHS data showed that 85% of pregnant women attend at least one antenatal visit, 70% of live births occurred in a health facility (97% in Kinshasa) and of children 12 to 23 months of age, 71%, 59%, 45% received the first, second and third doses of DPT immunization administered according to the WHO immunization schedule (20) and the DRC’s Expanded Program of Immunization at 6, 10, and 14 weeks respectively, while 63% had been vaccinated against measles (at 9 months). Moreover, breastfeeding is almost universally accepted (9 out of 10 children are still being breastfed at the age of one) in DR Congo. Yet, recent national surveys [5,6] showed that only 69% of 0 to 1 month old are exclusively breastfed while 65% of 2 to 3 month old are receiving something other than human milk in an environment where, according to the recent WHO/UNICEF progress report on sanitation and drinking-water [7], only 23% of the urban population have access to improved sanitation facilities and less than 50% to improved drinking-water sources.

Optimal breastfeeding practices, including immediate postpartum initiation of skin to skin contact with breastfeeding within one hour of birth, exclusive breastfeeding (EBF) with no additional fluid or food for 6 months [8], and continuation of breastfeeding thereafter up to 24 months and beyond with age appropriate complementary feeding, have great potential for reducing under five mortality rate [9-11]. If at least 90% of children were exclusively breastfed for the first six months of life, the potential reduction in mortality would be higher than from any other known effective intervention [10]. In most sub-Saharan countries, particularly in those with no or insufficient progress towards MDG 4, the prevalence of EBF among infant 6 months old or younger has not increased substantially and remains generally below 40% [12].

Starting in 1990, global initiatives to improve breastfeeding practices focused on maternity-level policies and practices known as the *Ten Steps to Successful Breastfeeding,* which serve as the basis for the *Baby-friendly Hospital Initiative* (BFHI) [13]. A maternity facility can be designated 'baby-friendly' when it has implemented the *Ten Steps* and has been reviewed using a national assessment approach. These steps include the following: (1) having a written breastfeeding policy that is routinely communicated to all healthcare staff, (2) training all healthcare staff in skills necessary to implement this policy, (3) informing all pregnant women about the benefits and management of breastfeeding, (4) helping mothers initiate breastfeeding within 30 minutes of birth, (5) showing mothers how to breastfeed and maintain lactation, even if they should be separated from their infants, (6) giving newborn infants no food or drink other than breast milk, unless medically indicated and not accepting free or low-cost breast milk substitutes, feeding bottles or teats, (7) allowing mothers and infants to remain together 24 hours a day, (8) encouraging breastfeeding on demand, (9) giving no artificial teats or pacifiers to breastfeeding infants, and (10) fostering the establishment of breastfeeding support groups and referring mothers to them upon discharge from the hospital or clinic. Implementation of the *Ten Steps* is associated with improvement in the rates of EBF [14-16].

BFHI is not being implemented to any extent today in DR Congo. The main attempt to implement BFHI steps in the country was led by UNICEF in the early 2000s as part of a national campaign of breastfeeding promotion, Overall 25 health facilities including 13 in Kinshasa out of more than 6,000 eligible facilities were certified through this effort. The last hospital certified was in 2004 in Katanga province when the funding stopped. Just two years after the peace deal that ended the deadliest war since World War II, which, in addition to decades of gross mis-management, have left the country infrastructures in shambles [17,18], the country was simply not ready to take over the initial UNICEF efforts.

Beside the fact that BFHI is not systematically implemented in DR Congo, to our knowledge, there has been no published report on breastfeeding practices and factors that are behind the persistent high rate of early supplementation. Data on West Africa as a whole indicates that water supplementation was a major contributor to the high rates of less than exclusive breastfeeding in the early 1990s, with most neighboring countries reporting more than 50% offering water in the first months of life. In 2000, only about 35% of women in DR Congo were still providing water to their infants in the first months of life [19,20], possibly accounting for the increase in EBF rates noted above.

This study was carried out as part of a preliminary phase of a cluster randomized trial designed to test whether support for the implementation the *Ten Steps,* coupled with provision of breastfeeding support in well-child and immunization clinics as a unique approach to the *Step* Ten (establishment of breastfeeding support groups in community), would improve the rate of EBF at six months in Kinshasa (NCT01428232). The main objective was to describe breastfeeding practices and identify the main determinants of the widespread early supplementation in Kinshasa, DR Congo.

## Methods

A cross-sectional survey was carried out in six selected health facilities offering maternal and child health services in Kinshasa, DR Congo. The six health facilities were selected from a network of 44 maternity clinics that receive support from The University of North Carolina at Chapel Hill in partnership with the Kinshasa School of Public Health for implementation activities to prevent transmission of HIV from mother-to-child. To inform selection, study staff (two medical doctors) visited all 44 facilities between October and November 2011 and collected information on factors that might affect the quality of care provided in each facility. Health facilities were then stratified by location (urban or periurban) and type of management. Within each stratum, facilities were sorted by the number of births and the proportion of mothers returning for one week postpartum visit and matched across strata for the average workload (number of births/number of personnel). Six facilities with relatively comparable workload across strata, the largest number of births per month, and the highest return for the one week visits within each stratum were selected. This selection scheme was adopted to ensure that health care facilities at the periphery of the city, which might have lower patient volumes with lower socio-economic status than facilities in urban areas, were included in the final sample while limiting the overall heterogeneity among the selected facilities.

In each facility, all healthcare personnel of the maternity and well-child clinics and a convenience sample of mothers of healthy infants six months or younger presenting consecutively at the well-child clinic who agreed to participate in the study, were recruited and interviewed. Interviews were administered by two trained and bilingual (French and Lingala, the main language used in Kinshasa) interviewers using structured questionnaires developed and adapted from Infant Feeding Practice Study II’s [21] and the Demographic and Health Survey’s [22] questionnaires. The mothers’ questionnaire was available in French and Lingala and contained questions on socio-demographic characteristics, maternity experience, breastfeeding support received, infant feeding practices (including a 24-hour recall), and other questions related to knowledge, beliefs and attitudes regarding breastfeeding. Healthcare worker’s questionnaire was available only in French (the official working language) and contained questions on professional experience, past training on breastfeeding, knowledge, attitude and practice of breastfeeding support, and questions related to the implementation of *Ten Steps* in their facility. Each questionnaire contained both open and closed ended questions and took about 30 to 45 minutes to administer.

Information collected with questionnaires was double-entered into an EPI-Info database. All responses to open ended questions that were given in Lingala were immediately translated into French prior to data entry by our bilingual study staff. The data was summarized using median and interquartile range (IQR) for continuous variables and proportions for categorical variables. All responses to open ended questions were reviewed for themes, and similar responses were grouped together in simple counts. All analyzes were conducted using SAS 9.2 (SAS Institute, Cary NC).

The study was approved by the Institutional Review Board of the University North Carolina at Chapel Hill (study # 11–1332) and the Ethical Committee of the Kinshasa School of Public Health. All participants provided signed informed consent.

## Results

### Characteristics of participants

#### Mothers

Ten mothers were interviewed in each of the 4 selected facilities, 15 and 11, respectively, from the two remaining facilities, for a total of 66 mothers. The median age of the mothers was 25 years (IQR 20 to 29) and that of their infant was 3 months (IQR 2 to 4). Over 80% were married or living with a partner (Table 1). Most mothers (86%) reported having completed more than the primary level of education. Only four mothers had a salaried (formal sector) job, while 73% worked as informal traders at home, in their neighborhood or at the market. The median number of children reported to be alive for each mother was 2 (IQR 1 to 3). Thirty-seven mothers (56%) reported having someone at home who helped to care for their infants. This help consisted of 14 in-laws, 2 maids, and 20 mothers, grandmothers, or other family relatives.

**Table 1:** Characteristics of study participants from a survey of mothers attending 6 well-child clinics in Kinshasa

	**Number**	**Percent**
Age in years: median (IQR)		25 (20. 3)
**Marital status**		
Single	12	18.2
Married	24	36.4
Live-in boyfriend	30	45.5
**Education**		
Primary	9	13.6
Secondary	49	74.2
University	8	12.1
**Occupation**		
Home/neighborhood small trade	37	56.1
Market small trade	11	16.7
Salaried (paid job)	4	6.1
Sewing	4	6.1
Hairdresser	7	10.6
Student	2	3.0
Farm work	1	1.5
**Number of surviving children:**		2 (1,3)
1	25	37.9
2	18	27.3
3	14	21.2
4+	9	13.7
**Previous child loss**		
Yes	3	4.5
No	63	95.5
**Child age in months:** median (IQR)		3 (2,4)
1	9	13.6
2	17	25.8
3	16	24.2
4	13	19.7
5	10	15.2
6	1	1.5

Abbreviation: *IQR* Interquartile range.

#### Healthcare workers and hospital environment

Forty-eight healthcare providers were also interviewed. They were predominantly female (42), nurses (35), midwives (11) and doctors (2). They had being working in their respective health facilities for an average of 14.2 years (range: 1 – 37 years). Nonetheless, only 8 reported having ever received any formal training on how to provide breastfeeding support to mothers. The training received was mainly as part of training on prevention of mother-to-child transmission of HIV (PMTCT); none of the trainings included a supervised practice section. No written breastfeeding policy was observed in the six facilities (or any of the 44 surveyed). However, all held regular health promotion classes during antenatal clinic visits in which breastfeeding was discussed. However, the information discussed was not recorded on antenatal clinic visit card or anywhere else. After a normal delivery, children and mothers were kept in the immediate postpartum observation room for few hours during which skin-to-skin contact was practiced. During the immediate postpartum period, infants and mothers were kept together in the same bed in the postpartum room for 2 to 3 days before they were discharged. Commercial infant food, artificial teats, and pacifiers were not observed in the delivery room or postpartum rooms. No advertisement for breast milk substitute was observed. Sugar water supplementation when given was with a spoon (usually) or syringe (drop in the mouth). For formula, families have to buy everything including bottles from the market.

### Breastfeeding practices

All 66 mothers were breastfeeding on the day of the interview. Twenty mothers (30%) reported initiating breastfeeding within the first hour after birth; 20 (31%) reported initially feeding their infants something else before breastfeeding initiation (Table 2). The early supplementation, prior to breastfeeding initiation, was mainly with sugar water for 16 (80%) infants, plain water for two, and formula for the last two others. A 24-hour recall of infant feeding showed that 26 (39%) infants were being exclusively breastfed. Of the exclusively breastfed infants, 19 (73%) were 3 months of age or younger. Supplements used in the last 24 hours included: water (15 infants), sugar water (1 infant), tea (2 infants), formula (12 infants), and porridge (10 infants). Liquid supplementation, such as sugar water, water or tea, dropped from 67% among one month olds to 25% among 3 month olds and 10% among 5 months old infants. All formula supplementation was among infants 4 months or younger, while 7 of the 10 infants supplemented with porridge were 4 months or older.

**Table 2:** Reported breastfeeding practices

	**Number**	**Percent**
Early initiation of breastfeeding**		
Yes	20	30.3
No	46	70.8
**Supplementation prior to breastfeeding initiation**		
Yes	20	31.3
No	44	68.8
**Supplements given prior to breastfeeding initiation**		
Sugar water	16	80.0
Water	2	9.5
formula	2	9.5
**Exclusive breastfeeding**		
Yes	26	39.4
No	40	62.1
**Supplements**		
Liquid (water, sugar water, tea)*	18	45.0
Formula	12	30.0
Porridge^¶^	10	25.0

**Initiation of breastfeeding within 1 hour of birth.

*Liquid supplementation only; ^¶^either with other liquid or alone.

### Mothers’ reasons for supplementing

Mothers who reported any supplementation were asked to state the reasons why they decided to give the particular supplement to their babies. The most common reasons for “liquid only” supplementation was hot weather and cultural beliefs that water is required for proper digestion of breast milk (Table 3): “***It was very hot****”* – 18 year old mother of a three months old infant; “***I have always heard that breast milk is hot***” - 27 year old mother of a one month old infant; “***breast milk is hot and water must be added for proper digestion***” – 23 year old mother of a six month old infant; “***Water is life! A child has to drink water***” – 20 year old mother of a four month infant. Water was also given as treatment for hiccups. For formula supplementation, the most frequent reasons given were that the baby was crying all the time (9/12), beliefs that the baby was not getting enough milk (5/12), or the infant was sucking all the time (4/12). One mother reported that it was because she wanted to go back to work/school. Porridge was mainly given as supplemental food when the mother or someone else thought it was the proper age: “***my stepmother asked me to start giving him porridge at 4 months***” – 20 year old mother of a five month old infant; “***my baby wasn’t getting full***” – 27 year old mother of five month old.

**Table 3:** Mothers’ reasons for starting the reported supplementation

Type of supplement and main reasons*	**Frequency**
**Liquid (water, sugar water, tea) alone**	**17**
Hot Weather: *“it was very hot”; “his lips were dry after feeding”*	6
Cultural beliefs: *“breast milk is hot and it's a meal! Water must be added”; “breast milk is hot! Water must be added for proper digestion”; “Water is life! the child must take water”; “He was having hiccups”*	6
*I thought I did not have enough milk*	5
**Formula**	**12**
*I thought I did not have enough milk*	5
*My baby was crying all the time*	9
*My baby was sucking all the time and it was difficult*	4
*The baby's father wanted it added to human milk*	2
*The grandmother of the baby wanted it added to human milk*	2
*Other*	3
**Porridge**	
*I thought I did not have enough milk*	1
*My baby was crying all the time*	5
*My baby was sucking all the time and it was difficult*	2
*The baby's father wanted it added to human milk*	0
*The grandmother of the baby wanted it added to human milk*	2
*Other*	3

Responses were elicited with the following question: Why did you decide to add (name the addition) to breast milk? Response options were provided and selection of more than one reason was allowed. Option other was provided for participant to specify reason that were not provided in the options.

### Knowledge, attitudes and beliefs

#### Mothers

Mothers’ questionnaire contained eight statements regarding breastfeeding benefits, establishment and maintenance of milk supply, and breast milk supplementation. These statements were read to mothers and they were asked to state for each whether it was “true”, “false”, or if they “don’t know”. In general, mothers appeared to have some knowledge of the benefits of breastfeeding for both themselves and their babies (Table 4). Over half (54%) of them agreed that “*supplemental feeding is detrimental to the establishment and maintenance of good milk supply*”; 45% agreed that “*a baby who is breastfed still needs to drink water like everyone else”.* Mothers were also asked to state what they believed was the recommended duration of exclusive breastfeeding. Only 36 (55%) mothers correctly stated six months while 29 (44%) stated a recommended duration between 1 and 5 months.

**Table 4:** Beliefs, attitudes, about breastfeeding and breastfeeding practices among mothers and health care workers

Knowledge about breastfeeding*	**True (%)**	**False (%)**	**Don’t know (%)**
**Mothers**			
*A woman who is exclusively breastfeeding is less likely to become pregnant three months after delivery than a woman who is formula feeding*	39 (60)	9 (14)	17 (26)
*Supplemental feeding is detrimental to the establishment of a good milk supply*	36 (55)	13 (20)	17 (26)
*It is usually advisable for babies to receive liquid (water, sugar, juice, etc.…) before the first breastfeeding*	8 (12)	47 (71)	11 (17)
*It is usually advisable for mothers not to give their babies the first milk that comes out after delivery*	6 (9)	50 (76)	10 (15)
*If a child who is breastfed has not regained his birth weight by two weeks, the mother should be encouraged to start supplement breast milk with the bottle.*	13 (20)	43 (65)	10 (15)
*A breastfeeding mother who feels that breast milk is insufficient should supplement it with a bottle or porridge after each feeding.*	33 (50)	28 (42)	5 (8)
*A baby who is breastfeed still needs to drink water like everyone else*	30 (45)	30 (45)	6 (9)
*If a baby cries at night, it is recommended to give him a bottle before putting him/her to sleep.*	7 (11)	55 (83)	4 (6)
**Health care workers**			
*In the first 1 or 2 days after birth the quantity of milk produced is too small to meet all the needs of a baby*	36 (75)	10 (25)	0
*Colostrum acts as a purgative and is important to clear the meconium and helps prevent jaundice*	42 (88)	3 (6)	3 (6)
*A newborn who is being breastfed still needs to drink water like everyone else*	-	48 (100)	-
*Children who are exclusively breastfed tend to have fewer episodes of diarrhea*	48 (100)	-	-
*Children who are exclusively breastfed are more likely to develop an ear infection or pneumonia*	-	47 (98)	1 (2)
*Regular and frequent breastfeeding can help reduce the risk of uterine bleeding and help the uterus return to its previous size*	44 (92)	3 (6)	1 (2)
*Frequent, on demand, and prolonged breastfeeding can help reduce the risk of breast or ovarian cancer*	32 (67)	6 (12)	10 (21)
*Mothers who think their breast milk is insufficient for their baby should top up each breastfeeding with a bottle of formula or porridge, juice etc.…*	35 (73)	2 (4)	11 (23)

*Statements were read to participants and they were asked to state for each whether it was “true”, “false”, or if they “don’t know.”

#### Healthcare providers

A modified version of the 8 statements was also used to evaluate the knowledge of healthcare workers. Overall, the healthcare workers appeared to have some knowledge of breastfeeding benefits. However, while all of them responded that it was false that “*a newborn who is being breastfed still needs to drink water like anyone else”* or that it was true that “*children who are exclusively breastfed ten to have fewer episode of diarrhea,”* 36 (75%) of them wrongly stated it was true that “*in the first 1 or 2 days after birth the quantity of milk produced is too small to meet all the needs of a baby*” and 35 (73%) that “*mothers who think that their breast milk is insufficient for their baby should top up each breastfeeding with a bottle of formula or porridge, juice etc. . .”* (Table 4).

### Breastfeeding support

#### In hospital

Only two mothers reported not attending any antenatal clinic (ANC) visit during their pregnancy. Of those who attended at least one ANC, 56 (86%) recalled that breastfeeding was discussed. However, only 33 (50%) mothers recalled a healthcare provider helping them with breastfeeding following the birth of their infants. The same number of mothers also recalled receiving some help during a well-child clinic visit while 20 (30%) reported receiving help from a health provider both in the maternity after birth and during well-child clinic visit. Mothers who reported receiving help from healthcare staff were asked in a follow-up question, what the staff did to show them how to breastfeed. Healthcare workers mainly showed them things about positioning, attachment, or nipple care. Not a single mother mentioned discussion of EBF or water supplementation. On a scale of “*0*” to “*5*” with “*0*” meaning “*Not at all useful*” and “*5*” meaning “*Very useful*”, all mothers scored the help they received “*3*” or above; with 22 (67%) given the maximum score - “*5*”. However, only three of the 48 healthcare workers interviewed reported that there was an actual plan in their facility to help mothers to breastfeed their infants.

#### In communities

Mothers were also asked if someone else showed them how to breastfeed after delivery beside hospital personnel. Of the 22 (34%) mothers who responded yes, 12 reported getting the help from their own mother or grandmother, five from their mother in-laws and the remaining from their sister (1), neighbor (1) or a nurse in the neighborhood (2). As with healthcare providers, the help reportedly focused mainly on child positioning and nipple care. Only one mother mentioned that she was informed by the person not to give any water or anything else to her baby.

Healthcare workers were asked if there was a breastfeeding support group to which they referred mothers at their discharge from the maternity clinic, all 48 healthcare providers responded no.

## Discussion

The goal of this study was to describe breastfeeding practices and identify potential determinants of the persistent low rate of EBF in Kinshasa. Using the Social Ecological framework [23], a conceptual model that addresses the importance of interventions directed at changing intrapersonal, interpersonal, organizational, community, and public policy factors which support and maintain unhealthy behaviors, our results suggest that the most important determinants of unnecessary liquid supplementation and early introduction of complementary food are mainly to be found in the first three levels of the framework: 1) the individual level characteristics such as knowledge, attitudes, beliefs, and skills; 2) the interpersonal factors, particularly the support from family, friends and healthcare providers; and 3) community factors such as community support groups or breastfeeding friendly hospital policy. The need for comprehensive support was also reflected in a recent review of community-based support for breastfeeding that concluded that interventions appear to have the greatest impact when they are part of multi-level, comprehensive interventions [24].

At the core of the widespread use of supplemental feedings is the limited knowledge among healthcare providers and mothers about human milk production, composition, and its adequacy for infants’ needs. This combined with the fact that over 73% of healthcare providers interviewed believed that the quantity of milk produced in the first 24 to 48 hours after birth is not sufficient for an infant’s needs with their expressed concern about low blood sugar, it is easy to understand why such a high number of infants are supplemented (in particular with sugar water) even before initiation of breastfeeding. Although concern about low blood sugar in newborns is justifiable, as it can cause brain injury [25], the best way to prevent low blood sugar in newborns is early initiation of breastfeeding with frequent feedings [26-28]. Moreover, recommendation or provision of sugar water to infants by healthcare workers contradicts the message on EBF and reinforces the cultural misbeliefs that “*an infant needs to drink water*”. In addition, of mothers who reported having received some breastfeeding support after birth or during well-child visits, the only somewhat consistent advice was on positioning, and nipple care. But it is unclear what nipple care might include that would not be potentially damaging.

Furthermore, it is of interest that despite the relatively high level of education among this group of urban mothers, up to 45% of them did not know the recommended duration of EBF. The fact that most mothers who gave solid food to their infants did so because of their belief that the child had reached the age to start complementary food, suggests that social desirability bias may be associated with either of the two responses. However, whether mothers knowingly gave a wrong recommended duration of EBF as a way of justifying their own introduction of complementary food early or because they did not know the recommended duration of EBF, may suggest that the knowledge and subsequent behaviors are modifiable with proper education and support.

Most mothers reported having someone at home who helps them take care of their infants and that this person has consider influence on how the infant is fed. Interventions aiming at changing some of the less than optimal breastfeeding behaviors should include an effort to reach and educate family members about EBF and its recommended duration. Half of the participating mothers reported receiving some breastfeeding support during well-child clinic visits suggesting well-child clinics could be used as potential alternative or supplement to community support for breastfeeding mother after discharge from maternity unit. But the effectiveness of well-child clinics as a unique approach for Step 10 merits further study.

Formula supplementation was observed mostly in the first three to four months of life. The primary reason given by mothers for formula supplementation was the impression that the baby was not getting enough milk. It is well documented that most mothers who stop exclusively breastfeeding do so because of the perception that their infant is not getting enough or is not satisfied with breast milk alone [29]. However, it is recognized that this ‘reason’ is often a proxy for other problems and constraints in the mother’s life [30]. The lack of readily available source of breastfeeding information and support following hospital discharge may explain, in part, the high frequency of formula supplementation [31,32]. Moreover, in addition to the non-implementation of the 10 steps to successful breastfeeding, the lack of training of health workers combined with the absence of a written policy on breastfeeding in health facilities, limits the impact of the observed efforts to implement other steps of the BFHI.

Finally, it is of interest in this setting, where nearly all women breastfeed initially, that there was strong emphasis on positioning rather that patterns of breastfeeding. This may be due to the fact that this is the emphasis in many training materials developed in the industrialized countries, where few women have the opportunity to observe breastfeeding or have help from their family or friends who are successful breastfeeders. It may be important to adjust the content of breastfeeding training for health professionals in these settings to be less about latch on and more about patterns over time.

Despite the systematic approach used to describe breastfeeding behaviors and determinants, this study has limitations. Our data came from a small convenience sample of mothers and healthcare providers from a very limited number of health facilities in Kinshasa, DR Congo. Educated mothers who exclusively breastfeed their infants for the recommended duration may cluster in certain health facilities that might be seen as offering better quality of services. Even with the careful considerations that went into the selection of facilities that served as study sites, the small number of facilities (six) selected may not represent the average health facility in Kinshasa. Moreover, the small sample size of healthcare workers and mothers interviewed does not allow statistical testing, In addition, information on breastfeeding practices and reasons for supplementation were all collected through face-to-face interviews and are susceptible to recall or social desirability bias. However, the fact that the rate of EBF in our sample is comparable to the one reported in national representative samples [5,6] suggests a limited impact of these potential biases and offers some confidence for applicability beyond the study sample.

## Conclusion

This work supports the understanding that comprehensive approaches, especially including Step 2, training of health workers, and Step 10 Community support, may lead to changes in poor hospital practices that reinforce negative cultural beliefs and sub-optimal practices among mothers. This, coupled with the lack of community support groups, may account for much of the persistent low rate of EBF in Kinshasa, DR Congo.

## Competing interests

The authors declare they have no conflicts of interests.

## Authors’ contributions

MY, ML, and FB conceived and designed the study. MY and JLC were responsible for data collection and quality. MY analyzed the data, and wrote the first draft of the manuscript. All authors contributed to final review and editing, including interpretation of results, and read and approved the text as submitted.
